# Age-based challenges to type 1 diabetes management in the pediatric population

**DOI:** 10.3389/fped.2024.1434276

**Published:** 2024-09-02

**Authors:** Yung-Yi Lan, Rujith Kovinthapillai, Andrzej Kędzia, Elżbieta Niechciał

**Affiliations:** Department of Pediatric Diabetes, Clinical Auxology and Obesity, Poznan University of Medical Sciences, Poznan, Poland

**Keywords:** age-based challenges, type 1 diabetes, management, glycemic control, developmental stages, self-management, children, adolescent

## Abstract

Type 1 diabetes is rising in the pediatric population, affecting approximately 1.2 million children and adolescents globally. Its complex pathogenesis involves the interaction between genetic predisposition and environmental factors, leading to T cell-mediated destruction of insulin-producing pancreatic beta-cells. This destruction results in insulin insufficiency and hyperglycemia. Hence, managing type 1 diabetes requires a comprehensive approach that includes various aspects such as blood glucose monitoring, insulin therapy, carbohydrate counting, caloric intake monitoring, considering family habits and food preferences, planning daily schedules, and incorporating physical activity. Children with type 1 diabetes encounter age-specific challenges in disease management that may exacerbate the risk of metabolic complications and adverse health outcomes. These risk factors may be neurological, physiological, behavioral, psychological, or social, complicate management and necessitate tailored approaches for effective care. Regardless of the age group, primary caregivers have a high responsibility to maintain optimal glycemic control, including monitoring diet, daily activity, and administering insulin. By reviewing research on the challenges faced by pediatric patients with type 1 diabetes, we summarized key insights aimed at developing targeted interventions and support systems that enhance diabetes management and improve health outcomes in this vulnerable population.

## Introduction

Type 1 diabetes (T1D), affecting 1.2 million children and adolescents younger than 20 years, is the predominant autoimmune endocrine disorder observed in the pediatric population, with a rising global occurrence that varies among different races, countries, and regions (1, 2). This condition results from an immune-mediated destruction of insulin-producing beta-cells in the pancreas, which leads to insulin insufficiency and hyperglycemia (3). Therefore, effectively managing T1D involves a multifaceted approach encompassing frequent glucose monitoring, insulin dosing, assessing carbohydrate and caloric intake, adjusting treatment according to family habits, food preferences, and daily schedule, and encouraging physical activity (4, 5).

A comprehensive approach to T1D care is essential for optimal glucose control and long-term survival (5, 6). Recent studies emphasize the long-term benefits of ensuring optimal blood glucose control during childhood and adolescence in preventing complications in adulthood (6). Effective T1D management in children relies heavily on self-management, which includes managing symptoms, treatment, and the physical and psychosocial impacts of the condition, as well as lifestyle adjustments (7). Thus, T1D treatment should be supported by structured diabetes education (4). T1D control in the pediatric population is influenced by factors such as growth and developmental stages, psychological attributes, co-existing diseases, family dynamics, and care outside of the home (8). Innovative technologies for managing T1D, ranging from blood glucose sensors, insulin pumps, and decision support tools, aid in maintaining target glucose levels (9). However, these technologies can be overwhelming for some patients, introducing obstacles such as increased treatment costs, visibility of devices, and intrusive alarms (10, 11). These factors raise the complexity of T1D care in the pediatric population, causing challenges in achieving good metabolic control (8, 10, 11). This review comprehensively examines the unique obstacles faced by individuals with T1D at different stages of development from early childhood to adolescence.

## Data sources and searches

This narrative review did not involve a systematic literature search; each author identified and critically reviewed the most relevant papers. The work presents several studies on age-based challenges to T1D management in the pediatric population. The following electronic databases were searched for relevant full-text articles in the English language: PubMed, Google Scholar, Scopus, EMBASE, and Web of Science. The search time was from October 2023 up to May 2024 using the following keywords: type 1 diabetes; management; treatment; regimen; pediatric diabetes care, age-based challenges; infants; toddlers; preschoolers; early-elementary school-age children; school-aged children; adolescents; youth; children; transitional age; transition into adult diabetes care; developmental stages; limited cognitive capacities; communicational skills; autonomous behaviors; risky behaviors; glycemic control; self-management; awareness of hypoglycemia, signs of hyperglycemia; parental distress; parental burnout; type 1 diabetes burnout; adherence; non-adherence; compliance; drug use; alcohol use; family dynamics; financial; practical issues; social support; lifestyle; school; diabetes education; teachers. All articles published between July 2004 and May 2024 were checked by title, abstract, and full text. It aimed to detect the most clinically significant papers related to the topic and provide a theoretical point of view, which is considered a valuable educational tool in continuing medical education.

## Infants and toddlers

Managing T1D in younger children, particularly infants and toddlers, is troublesome due to their limited expressive language skills, making it difficult for them to reliably detect and report early signs of hypoglycemia (6). Additionally, a frequent feeding pattern, typical for this age group, may pose difficulties in T1D management, causing fluctuation in glucose levels between hyperglycemia and hypoglycemia (12).

During infancy, cognitive capacities hinder the understanding of the intricate aspects of T1D management. Consequently, the responsibility for disease management squarely rests on the shoulders of caregivers, underscoring their primary obligation to actively engage in a comprehensive T1D management plan (12, 13). Essential developmental milestones in infancy include establishing a bond with primary caregivers. Disruption of this process may lead to an underdeveloped trusting relationship between the infant and the caregiver. This impaired relationship is likely to complicate adherence to recommended Diabetes Self-Management Education (DSME). The DSME advocates for caregivers to ensure warmth and comfort following invasive procedures, such as replacing insulin pumps, establishing feeding and sleep routines, and maintaining vigilance for hypoglycemia, which are pivotal aspects of pediatric care (12). The presence of a distrusting and uncooperative child further exacerbates these challenges and fails to promote a supportive environment for both the infant and the caregivers.

As children progress into the toddler phase, a significant shift toward autonomy occurs, reflecting a natural strive for independence and control (14). However, this newfound autonomy can create challenges during feeding, as many toddlers exhibit picky eating behaviors and resist consuming offered food (15). This autonomy may negatively impact T1D management by causing significant fluctuations in blood glucose levels. Picky eating in children with T1D can lead to blood glucose fluctuations due to inconsistent carbohydrate intake and the potential for skipping meals. Favoring high-glycemic foods can cause rapid spikes and drops in blood glucose levels. These dietary inconsistencies complicate accurate insulin dosing, further contributing to glycemic variability. As a result, effectively managing a picky eater involves implementing limit-setting strategies and addressing the challenges associated with a toddler's non-cooperation with established routines (12, 14).

Hence, caregivers are compelled to adapt their parenting practices and integrate various daily tasks to enhance the quality of life for infants and toddlers managing T1D. Consequently, approximately one in five parents experience psychological distress within the initial four years following their child's diabetes diagnosis (16). Furthermore, managing T1D introduces significant changes in familial dynamics, causing notable transformations in both the parent-child relationship and the dynamics between partners (17). These exceptional challenges in caring for young children with diabetes contribute to heightened parental concerns and an increased impact on overall family well-being (17).

## Preschoolers and early-elementary school aged children

Treating T1D in the preschool phase and early elementary school-aged children involves exceptional complexities, requiring understanding a confluence of physiological, developmental, and psychosocial factors (18). These multifaceted concerns necessitate tailored interventions that address both the child's medical and emotional needs as well as parental distress and family burden.

Similarly, in early childhood, the ability to recognize and communicate symptoms of hypoglycemia may be limited (18). Moreover, parents may struggle to distinguish between behavioral signals of hypoglycemia or hyperglycemia and typical developmental behaviors, such as temper tantrums, lingering at mealtime, and refusing to eat presented food (15). Parenting dilemmas may arise concerning appropriate disciplinary measures for children with T1D, particularly in balancing an understanding of their condition while fostering accountability for their actions. Some parents justify a lenient approach by acknowledging the significant burdens already faced by children with T1D, believing these children already face considerable challenges (19).

Another issue in managing T1D is balancing physical activity with insulin dosing and food intake. Some data show that children diagnosed with T1D before the age of 7 often demonstrate markedly lower levels of physical activity compared to their healthy counterparts of the same age and gender (20). This decrease can be attributed to a variety of factors. Parents may refrain from encouraging physical activity for their young children due to concerns about hypoglycemia, or they may fail to adjust their child's diabetes management effectively in response to changes in physical activity levels (21, 22). The ramifications of diminished physical activity may result in heightened insulin resistance and an elevated susceptibility to complications associated with T1D, such as cardiovascular disease.

Parents often face challenging decisions regarding employment due to the demanding nature of caring for a child with T1D (19). Research by Harrington et al. (23) highlighted that 55% of parents require the need for flexible work arrangements, adversely affecting family dynamics. Specifically, 57% of parents express concerns about their child's well-being while away from home, especially as they spend more time in kindergarten. Another study showed that 44% of parents reported their child's T1D diagnosis influenced school enrollment decisions, and 12% had withdrawn their child from school or daycare due to difficulties managing the condition (24). Parents of younger children were particularly apprehensive about a school's ability to manage T1D effectively.

## School-aged children

School-aged children with T1D face significant troubles when assuming new responsibilities for managing their condition. Despite their potential capabilities, many rely heavily on parental assistance for various diabetes-related tasks. This dependence may stem from a desire to take breaks from daily management duties, engage in leisure activities, or avoid potential errors (25). Additionally, children often struggle with complex tasks such as carbohydrate counting and insulin dosing, further reinforcing their reliance on adults for support. This lack of proficiency not only impacts their daily activities but also restricts their participation in social events like sleepovers or school trips. These barriers highlight the need for tailored support and education initiatives to empower school-aged children with T1D to manage their condition effectively (26). Furthermore, children may experience negative emotions, including anger, fear, and sadness, due to the impact of their condition on social life and independence, with strict dietary restrictions being a significant source of dissatisfaction (27). Hence, they are at a higher risk of depression compared to their healthy peers. Symptoms of depression may manifest as irritability, mood changes, sleep or appetite disturbances, loss of interest in activities, decreased school performance, poor diabetes management, and feelings of being overwhelmed. Unfortunately, parents may misinterpret these signs as laziness or indifference rather than recognizing them as signs of depression. Childhood depression not only diminishes the child's quality of life but also correlates with poorer T1D control and increased instances of diabetic ketoacidosis (28).

As highlighted in various studies, the lack of information and training about T1D among teachers and classmates is a significant issue (1). Nearly half of the interviewed teachers were tasked with instructing students with T1D, yet only a few have undergone targeted training. This knowledge gap impairs their ability to effectively manage the condition, including monitoring blood sugar levels, administering insulin, and responding to emergencies (29). The need for specialized healthcare professionals, such as school nurses, underscores the reliance on external support systems to meet the needs of students with T1D, which may result in delayed or inadequate care during emergencies (30).

## Adolescents

Enhanced risk-taking behavior during adolescence, marked by increased experimentation with drugs and alcohol, accidental deaths, and unprotected sexual activity, has been extensively documented in studies (31, 32). For individuals with T1D, substance use is especially problematic in that it increases the likelihood of acute complications and hospitalization. Alcohol inhibits gluconeogenesis and glycogenolysis in the liver, leading to decreased blood glucose levels for several hours after consumption, which can be life-threatening when combined with insulin therapy (33). Additionally, alcohol intoxication impairs cognitive function, which may result in missed insulin doses, miscalculated dosages, or delayed blood glucose monitoring, further exacerbating the risk of hypoglycemia. Therefore, it is crucial for adolescents to be aware of these risks, regularly screened for alcohol intake, and provided with appropriate education (34, 35).

Drugs can worsen hypoglycemia unawareness because their symptoms often mimic those of hypoglycemia. As a result, both the person with T1D and those around them may mistake signs of hypoglycemia for symptoms of intoxication. A hypoglycemic event that is not recognized and treated quickly can result in loss of consciousness and/or seizure, necessitating more intensive care and increasing morbidity and mortality (36–38).

Adolescents with T1D are significantly more likely to develop eating disorders (ED), with research showing a 2.5-fold increased risk compared to their healthy peers (39). For every occurrence of ED in healthy teenagers, approximately two to three adolescents with T1D are similarly affected. Key factors contributing to this comorbidity include elevated body mass index (BMI), recurrent weight fluctuations due to frequent dieting, dissatisfaction with body image, depressive symptoms, and ineffective coping mechanisms (40, 41). Adolescents with T1D may turn to insulin restriction to achieve their desired body weight, driven by factors such as peer influence, normalization of unhealthy behaviors, and underlying mental health issues such as depression and anxiety. Addressing these multifaceted factors is crucial for interventions aimed at promoting healthy weight management and overall well-being in this population (42–46).

Disease perceptions, encompassing individuals’ beliefs and understanding of their condition, have been shown to influence the management of T1D in adolescents significantly. Negative perceptions may lead to reluctance to engage in essential self-care behaviors, causing fluctuations in glucose levels and increasing the risk of complications. Furthermore, such perceptions may deter individuals from seeking necessary healthcare support, exacerbating difficulties in management and increasing the likelihood of adverse health outcomes (47–49).

Peer support is crucial in managing T1D, as it offers adolescents a comforting outlet to share their experiences. Adolescents with T1D often grapple with a spectrum of negative emotions like anger, fear, and distress stemming from the perceived intrusion of the condition into their lives or from perceiving T1D as a barrier to “normal” life (27, 50). These feelings contribute to resistance in adhering to management routines, exacerbated by resentment towards the condition, especially regarding its impact on social life and independence. Concerns about peer perceptions may trigger social withdrawal and avoidance of activities, worsening emotional distress (51, 52).

## Transition age

**Figure 1:**
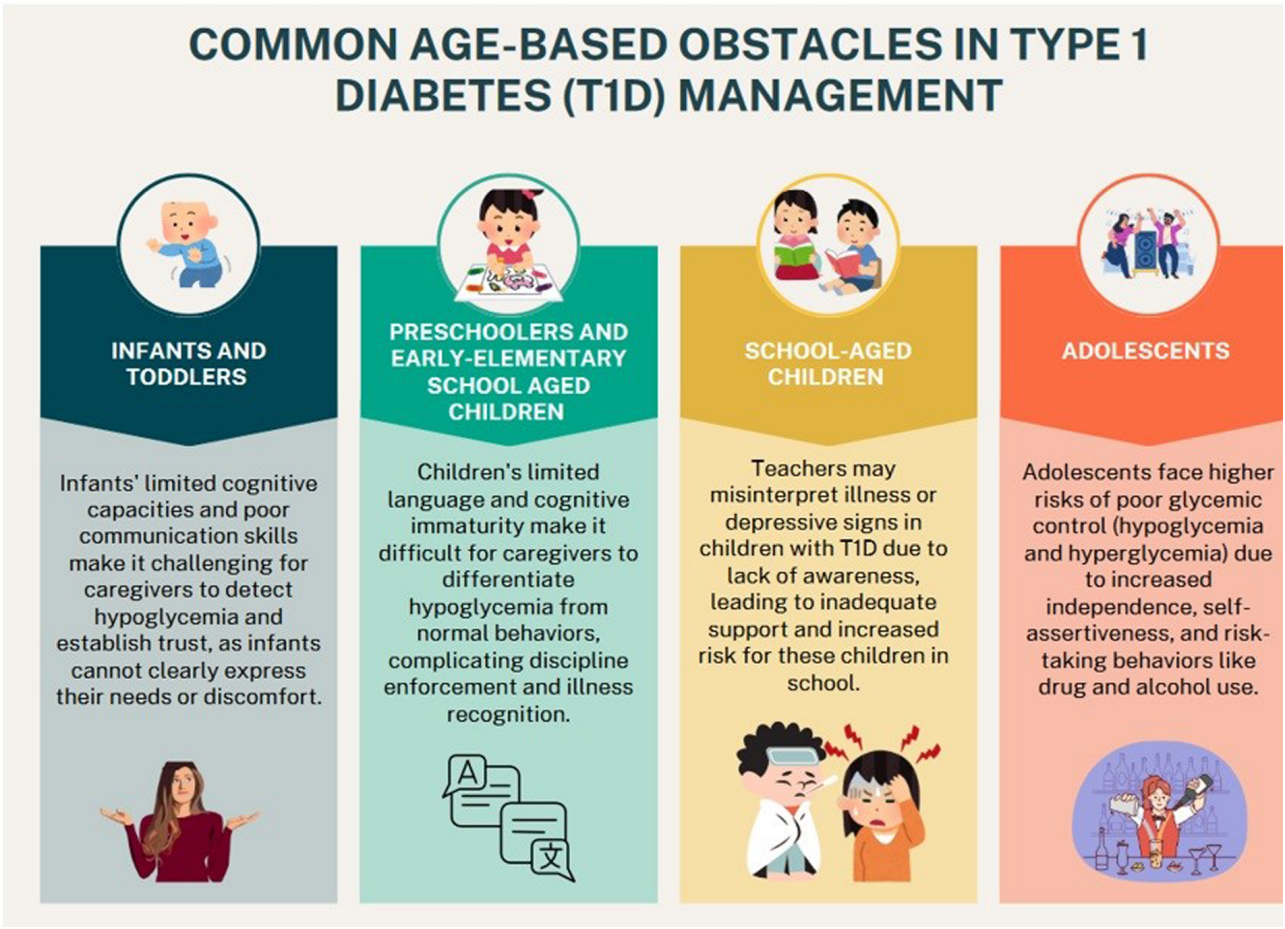
The main age-based obstacles in type 1 diabetes management in the pediatric population.

Transitioning from adolescence to adulthood presents numerous challenges in the management of T1D. Mistry et al. (53) reported that 32% of adolescents fail to attend a scheduled appointment within a year after their transition from pediatric to adult clinics, exemplifying the need for tailored support during this critical period. Some findings suggested that those diagnosed with T1D at a younger age may face difficulties in transitioning and managing T1D into adulthood, potentially due to less autonomy in T1D management as parental involvement declines (53). Ladd et al. (54), however, concluded that being diagnosed at a young age and having a longer T1D duration prepared adolescents for better adjustment and transitioning of self-management compared to those diagnosed late in adolescence. The lack of preparation for independent management, as well as insufficient engagement, is compounded by parental endorsement of attending all diabetes clinic visits, with the majority admitting to not having initiated discussions regarding transition plans with their adolescent (65%) or the diabetes care team (60%) (55). Some adolescents regard the transition process as daunting, expressing a desire for developmentally appropriate interactions and meaningful conversations about diabetes care. Social support and coordination between pediatric and adult care providers are crucial for ensuring a smooth transition and mitigating the risk of complications later in life. This highlights the importance of identifying high-risk patients and providing individualized support to facilitate successful transitions and long-term disease management. Table 1 summarizes the challenges encountered by individuals with T1D and their caregivers, along with proposed solutions regarding the management, while Figure 1 highlights the main age-based obstacles in T1D management in the pediatric population.

**Table 1:** Summary of challenges encountered by individuals with T1D and their caregivers with proposed solutions.

Age group	Potential obstacles in T1D management in patients	Potential obstacles in T1D management in caregivers	Solutions
Infants and toddlers	– Limited cognitive capacities	– Unrecognized hypoglycemia	– Structured T1D education for family
	– Poor communication skills	– Establishing trust with a child	– Integrate user-friendly technological solutions
	– Lack of cooperation	– Following healthy eating habits	– Provide emotional support and counseling services
	– Autonomous behaviors	– Psychological distress/burnout	
		– Adverse effects on family	
Preschool and early elementary children	– Limited language/cognitive function/ability to express symptoms	– Enforcing disciplinary measures	– Implement behavioral interventions to address picky eating
	– Exhibit developmental behaviors	– Distinguishing between hypoglycemia and typical developmental behaviors	– Develop individualized diabetes management plans with schools
	– Increase need for physical activities and energy expenditure	– Concerns about hypoglycemia restricting physical activity	– Offer parental support programs
		– Negative impact on employment, financial strain, family dynamics	– Provide age-appropriate educational materials
School-aged children	– Dependence on caregivers for T1D management	– Misinterpretation of signs of illness or depressive behaviors	– Implement peer education programs in schools about T1D, reducing stigma
	– Struggle with carbohydrate counting and insulin dosing	– Lack of T1D awareness and specialized training among teachers	– Provide comprehensive training for teachers to recognize and respond appropriately to hypo/hyperglycemia – Foster gradual independence among children with T1D
	– Increased vulnerability within the school environment	– Failure to assist and provide support during emergencies or situations requiring immediate attention	– Ensure access to trained health professionals like school nurses
	– Non-adherence to healthy behaviors exhibit negative emotions		
	– Increased risk of T1D complications		
Adolescents	– Increased risk-taking behaviors	– Increased risk-taking behaviors, peer influences	– Manage risk-taking behaviors/Foster peer support and social engagement
	– Psychological changes	– Lack of proper balance between parental involvement and adolescent autonomy	– Acknowledge psychological factors and prioritize mental well-being
	– Higher risk of developing eating disorders	– Absence of discussions on transition to adult diabetes care	– Instill healthy eating habits and weight management
	– Negative disease and peer perceptions	– Insufficient social support and coordination between pediatric and adult care providers	– Enhance disease perceptions and encourage self-care behaviors
	– Difficulty transitioning from pediatric to adult care		– Support adolescents to take ownership of their health

T1D, type 1 diabetes.

## The strengths and limitations of the review

This narrative review offers a practical and detailed examination of age-based challenges in managing T1D among pediatric populations, enhancing the existing pediatric diabetes guidelines by addressing specific obstacles encountered at different developmental stages. The review integrates a broad spectrum of studies to highlight the complex interplay of physical, psychological, social, and environmental factors influencing T1D management from infancy through adolescence. It provides actionable strategies and tailored interventions that complement general recommendations in current guidelines, offering a nuanced perspective on addressing the unique needs of young patients and their caregivers.

However, as a narrative review rather than a systematic review, it has certain limitations. It may not cover all relevant issues or include every pertinent study, potentially leaving out some aspects of the complex management of T1D. The review's findings are based on selected literature and expert opinion, which may not fully represent the breadth of evidence available in comprehensive guidelines. Consequently, while the review provides valuable insights and practical solutions, it should be considered alongside more exhaustive systematic reviews and clinical practice standards for a complete understanding of pediatric T1D management.

## Conclusions

In conclusion, managing T1D in pediatric populations requires a comprehensive approach tailored to the unique challenges encountered at different developmental stages. From infancy through adolescence, individuals with T1D and their caregivers face diverse obstacles related to physical, psychological, social, and environmental factors. Integrating user-friendly technological solutions, providing caregiver education and support, addressing picky eating behaviors, collaborating with schools, offering peer support programs, and facilitating the transition to adult care are crucial strategies. By implementing these interventions, healthcare providers and support systems can empower pediatric patients with T1D to thrive despite their condition, leading to improved long-term outcomes and quality of life.
